# *Phytophthora capsici*-tomato interaction features dramatic shifts in gene expression associated with a hemi-biotrophic lifestyle

**DOI:** 10.1186/gb-2013-14-6-r63

**Published:** 2013-06-25

**Authors:** Julietta Jupe, Remco Stam, Andrew JM Howden, Jenny A Morris, Runxuan Zhang, Pete E Hedley, Edgar Huitema

**Affiliations:** 1 Division of Plant Sciences, University of Dundee, Dundee DD2 5DA, UK; 2 Dundee Effector Consortium, James Hutton Institute, Invergowrie, Dundee DD2 5DA, UK; 3 Cell and Molecular Sciences, James Hutton Institute, Invergowrie, Dundee DD2 5DA, UK; 4 Information and Computational Sciences, James Hutton Institute, Invergowrie, Dundee DD2 5DA, UK

## Abstract

**Background:**

Plant-microbe interactions feature complex signal interplay between pathogens and their hosts. *Phytophthora *species comprise a destructive group of fungus-like plant pathogens, collectively affecting a wide range of plants important to agriculture and natural ecosystems. Despite the availability of genome sequences of both hosts and microbes, little is known about the signal interplay between them during infection. In particular, accurate descriptions of coordinate relationships between host and microbe transcriptional programs are lacking.

**Results:**

Here, we explore the molecular interaction between the hemi-biotrophic broad host range pathogen *Phytophthora capsici *and tomato. Infection assays and use of a composite microarray allowed us to unveil distinct changes in both *P. capsici *and tomato transcriptomes, associated with biotrophy and the subsequent switch to necrotrophy. These included two distinct transcriptional changes associated with early infection and the biotrophy to necrotrophy transition that may contribute to infection and completion of the *P. capsici *lifecycle

**Conclusions:**

Our results suggest dynamic but highly regulated transcriptional programming in both host and pathogen that underpin *P. capsici *disease and hemi-biotrophy. Dynamic expression changes of both effector-coding genes and host factors involved in immunity, suggests modulation of host immune signaling by both host and pathogen. With new unprecedented detail on transcriptional reprogramming, we can now explore the coordinate relationships that drive host-microbe interactions and the basic processes that underpin pathogen lifestyles. Deliberate alteration of lifestyle-associated transcriptional changes may allow prevention or perhaps disruption of hemi-biotrophic disease cycles and limit damage caused by epidemics.

## Background

Plant-pathogen interactions exhibit a dynamic interplay between host defense mechanisms and specialized pathogen structures that aim to subvert immunity. Although plants lack an adaptive immune system, they carry pattern recognition receptors (PRRs) that recognize microbe or pathogen-associated molecular patterns (MAMPs or PAMPs) and initiate effective defense responses. This form of innate immunity, termed PAMP-triggered immunity (PTI), ensures an early response to a broad range of potential pathogens, and generates systemic signals that travel to healthy tissues and prime defense signaling networks [1]. To counter plant defenses, pathogens deploy repertoires of secreted molecules (effectors) that, upon delivery into the host apoplast (extracellular effectors) or cell cytoplasm (intracellular effectors), modify cellular targets to suppress PTI and enable parasitic infection and reproduction [2-4]. In addition, pathogens may also secrete classes of effectors that provoke execution of host cellular processes required for disease development. Consequently, both host and microbe tightly control transcriptional programs that drive responses to external signals.

*Phytophthora *species are a destructive group of filamentous plant pathogens, which have a global distribution and devastating effect on a wide range of plants important to agriculture and natural ecosystems [5]. For example, *Phytophthora infestans*, the causal agent of late blight in potato and tomato crops, and *Phytophthora capsici*, an important pathogen of tomato, other solanaceous, and cucurbit plants, cause multi-billion dollar losses in crop production annually [6,7]. The economic impact of this group of pathogens remains the principal driving force behind the need to understand *Phytophthora *parasitism and epidemics. *Phytophthora *spp. are hemi-biotrophic pathogens, having a lifestyle that features a biotrophic phase, followed by a switch to necrotrophy [8-10]. This lifestyle is also common to other detrimental filamentous plant pathogens such as fungi that fall into *Magnaporthe*, *Colletotrichum*, and *Mycosphaerella *genera [11]. In the early biotrophic phase, specialized infection structures, termed haustoria, are formed to breach the plant cell walls and interface with the host membrane [12,13]. The initial biotrophic phase is crucial for infection and disease establishment, after which rapid intercellular growth and colonization occurs, ultimately leading to host cell death, sporulation, and initiation of a new infection cycle. Despite the availability of multiple *Phytophthora *genome sequences [7,14,15], little is known about the signal interplay between organisms that occurs during infection, or which processes in the plant contribute to the *Phytophthora *hemi-biotrophic lifestyle.

Throughout the infection cycle, *Phytophthora *secretes effectors into its host with the aim to promote pathogen growth and reproduction [16-18]. The *Phytophthora *effector repertoire consists of extracellular proteins (apoplastic effectors) that inhibit or counter defense-associated compounds and lytic enzymes, as well as classes of secreted proteins that traverse the host membrane and target intracellular processes (intracellular effectors) [19]. Of the intracellular effectors, the RXLRs, named after their RXLR-dEER amino acid motif, translocate across the haustorial host-pathogen interface, where they are thought to perturb host cellular signaling and suppress immunity [13,20,21]. Besides the RXLRs, *Phytophthora *genomes encode another large class of intracellular effectors, termed crinklers (CRN), which feature a conserved LFLAK motif that is required for effector translocation. These effectors exclusively target the host nucleus upon delivery [14,22,23].

Owing to the tremendous economic impact *Phytophthora*-host associations have on crop production, it is crucial to understand how this group of pathogens manipulates their host and promote damage. Current breeding strategies rely on introgression of resistance genes and identifying new R-gene variants [24-26], which are often rapidly overcome by the pathogen [27-29]. Therefore, increasing interest lies in the mechanisms underpinning infection, disease establishment, and epidemics. One strategy for studying these processes is to examine host and pathogen gene-expression patterns during the course of infection. This will enable the identification of essential transcriptional changes that occur in the pathogen during infection, its establishment, and the transition from biotrophy to necrotrophy. Equally important for understanding disease is identification of those host processes and signaling pathways that are perturbed by the pathogen while it progresses through its specific life stages.

In the current study, we explored the association between the broad host range pathogen *Phytophthora capsici *and tomato. Infection time-course assays reveal a distinct hemi-biotrophic infection cycle, featuring haustoria formation early in, infection, followed by necrotrophy in the late disease stages. We exploited the availability of genome sequences for *P. capsici *and tomato to measure gene-expression changes in both *P. capsici *and tomato simultaneously during the course of infection. Microarray analyses, using a custom-designed combined pathogen and host whole-genome array helped define transcriptional changes in *Phytophthora *that were linked to (disease) development, and identified distinct transcriptional responses in tomato, associated with pathogen lifestyle. These unveil a requirement for *Phytophthora *to have enhanced protein production and metabolism in biotrophy, catabolism during its transition to necrotrophy, and induction of signaling and developmental processes upon sporulation. *P. capsici *infection of tomato results in two dramatic changes in the host transcriptome, favoring defense signaling and metabolism early after infection, whereas during the transition from biotrophy to necrotrophy, genes required for (a)biotic stress, signaling, and regulation are activated. We hypothesize that these changes are driven by differentially regulated and stage-specific effector genes identified in our study.

Our results provide unique detail about the coordinated transcriptional reprogramming in both host and pathogen during infection, and lay a foundation for future studies on transcriptional programs that drive parasitic lifestyles. This work opens the door towards comparative transcriptomics studies that should help unravel pathogen infection strategies and exploit host basal defense responses.

## Results

### *P. capsici*-tomato interactions feature an early biotrophic and late necrotrophic phase.

**Figure 1:**
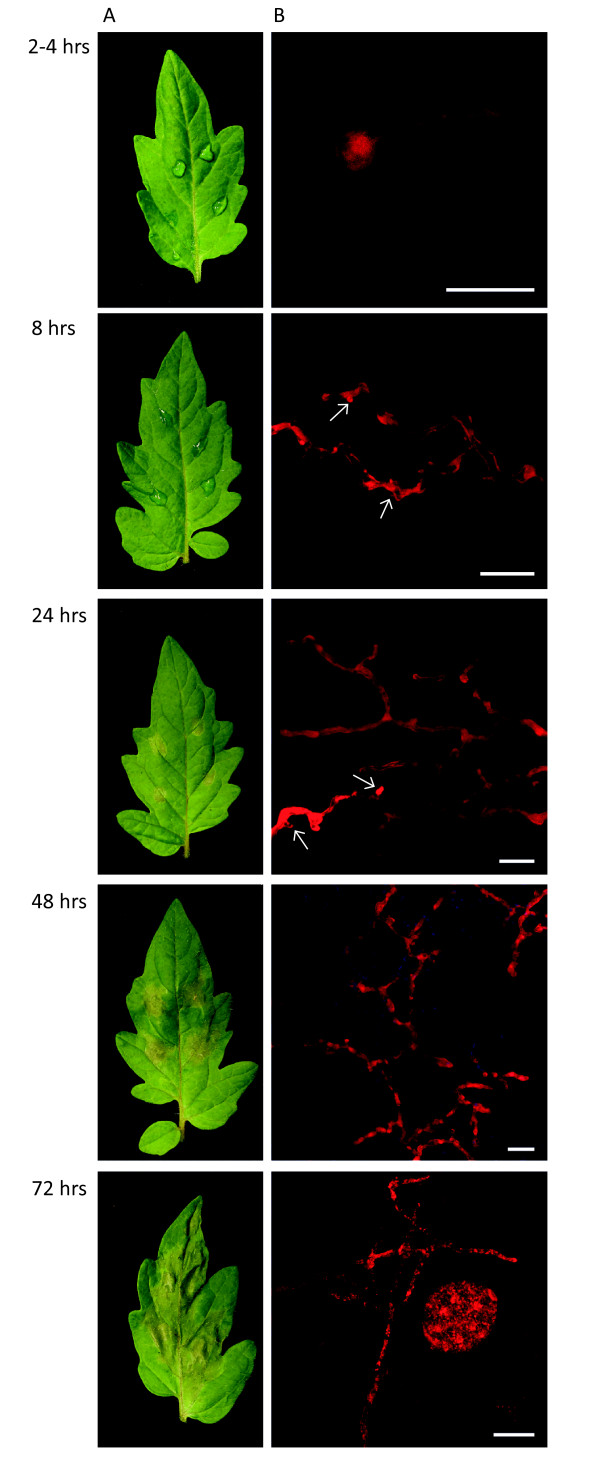
***Phytophthora capsici *infection of tomato features a hemi-biotrophic lifecycle**. **(A) **Tomato leaves infected with zoospore suspensions of *P. capsici *at 2-4, 8, 24, 48, and 72 hours post-infection (hpi). **(B) **Confocal microscopy images of tomato leaves infected with a transgenic *P. capsici *strain expressing the fluorescent protein TdTomato (red). Infection featured rapid germination of cysts and infection at 8 hpi, formation of biotrophy associated haustoria (arrowheads) visible up to 48 hpi after infection, and rapid growth and sporulation at 48 and 72 hpi respectively. Scale bar = 20 μm.

We investigated the interaction between *P. capsici *strain LT1534 and tomato (*Solanum lycopersicum *'Moneymaker') in time-course experiments (Figure 1). Inoculations followed by phenotypic analyses across time points suggested that in the early stages of infection (up to 24 hours post-infection (hpi)), *P. capsici *ingress features a biotrophic phase during which host tissues appear healthy and unaffected, followed by a necrotrophic phase (>24 hpi), marked by tissue collapse (Figure 1A). Multiple inoculation experiments showed distinct phenotypic changes in the later stages of infection, which included host tissue water-soaking, cell death, and tissue collapse (Figure 1A).

Based on these observations, we investigated whether *P. capsici *forms haustoria *in planta *and inoculated tomato plants. Zoospores derived from a transgenic *P. capsici *strain were used, which expressed the red fluorescent protein-coding gene *tdTomato*, and infection was monitored through confocal microscopy (Figure 1B). We observed germinating cysts as early as 1 hpi, and found germinating cysts with hyphae penetrating into the plant cells at 8 hpi (Figure 1B). Infection and subsequent colonization of leaf tissue was evidenced by growth of red fluorescent *P. capsici *mycelia in inoculated host tissues, and the formation of distinct haustorial structures at the early time points (Figure 1B). Confocal microscopy of leaf tissues in the late infection stages showed significant colonization of tissues with formation of sporangia at 72 hpi (Figure 1B). To assess whether cells were viable, we also inoculated transgenic *Nicotiana benthamiana *plants expressing ER-eGFP (associated with the endoplasmic reticulum; ER-eGFP) and assessed cell viability at relevant time points at drop inoculation sites (see Additional file 10: Figure S1). These analyses identified haustoria in living cells and low levels of cell death in the early phase (0 to 16 hpi), with increasing numbers of dead cells at 24 and 48 hpi (see Additional file 10: Figure S1). After tissue collapse at inoculation sites, living haustoriated cells were commonly seen at lesion edges, suggesting a dynamic infection cycle in which phase transition is separated spatially. These results are consistent with a hemi-biotrophic infection cycle and further confirm the presence of distinct developmental stages accompanying tomato infection.

### A composite host-pathogen microarray approach to simultaneously profile transcriptional changes during the *P. capsici*-tomato interaction

**Figure 2:**
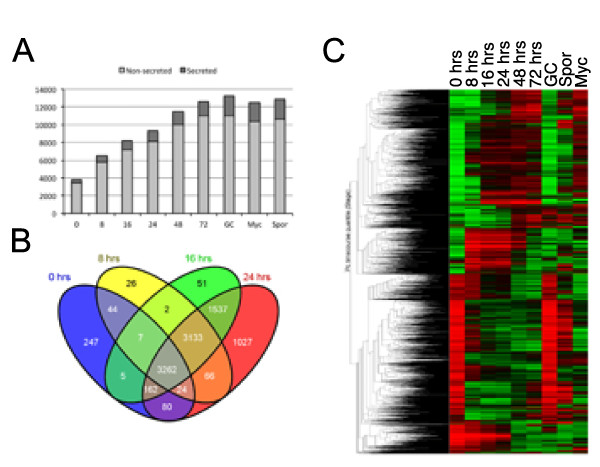
**Expression of *Phytophthora capsici *gene complement during infection and disease progression**. **(A) **Overview of genes that were expressed as detected on the *P. capsici*-tomato two-genome microarray. The proportion of genes encoding putative secreted proteins (effectors) are indicated by dark grey. **(B) **Assessment of overlap of genes expressed in infectious stages and **(C) **overall assessment of differentially expressed *P. capsici *genes, determined by ANOVA as described in the text. Red and green represent upregulated and downregulated genes respectively. The *y*-axis shows average linkage of Pearson correlations of gene-expression profiles. The Venn diagram was generated using Venny [49].

Considering that PTI features a shift in gene expression and cellular activity towards defense, and that pathogen effectors act to modulate defense gene induction, simultaneous profiling of the gene expression of both pathogen and host should help deduce coordinated relationships between transcriptional programs in host and pathogen. To understand the processes underpinning both *P. capsici *infection and disease progression in tomato, we designed a custom microarray (Agilent Technologies, Inc., Santa Clara, CA, USA) with 60-mer oligonucleotide probes to all gene models of *P. capsici *and *S. lycopersicum *[23], and measured gene-expression changes in both organisms across the same time-course infection experiment. Sites on detached tomato leaves that had been drop-inoculated with *P. capsici *strain LT1534 were harvested at 0, 8, 16, 24, 48 and 72 hpi (Figure 2). In addition to the infectious stages, samples were taken from tomato leaves that had been mock-inoculated with water (designated 'Non-infected tissue': Ni) and harvested at 0 hpi. Further sporangia and zoospores (taken at 0 hpi), germinating cysts (taken at 16 hpi), and mycelia grown *in vitro *(harvested at 48 hpi), were collected directly from the inoculum after the various incubation times. This experiment was repeated two times to generate three fully independent biological replicates for analysis.

*P. capsici *gene-expression analyses showed that of the 20,530 gene models represented on the array, 15,430 (75%) were expressed in at least one of the 6 infection and 3 *in vitro *stages sampled (Figure 2A,B). In each of the stages, a significant fraction of expressed genes encoded secreted proteins, ranging from 10.4% to 12.6% during all stages of infection and up to 17.4 % in the *in vitro *stages (Figure 2A; see Additional file 1: Table S1). Given the dynamic nature of pathogen infection and development, we assessed the expression patterns of *P. capsici *genes, and found large suites of genes that are specifically expressed in early stages of infection and throughout the infection process (Figure 2B). Differences in expression patterns were not solely due to low levels of detection in the early stages of infection, as distinct sets of genes, expressed only in these stages, were identified (Figure 2B). Subsequent statistical analyses identified 3,691 differentially expressed genes (one-way ANOVA, using Benjamini and Hochberg multiple testing correction, *P*≤0.005), suggesting dramatic transcriptional changes throughout the infection and growth process of *P. capsici *(Figure 2C).

### *P. capsici *shows defined shifts in gene expression during specific life stages

**Figure 3:**
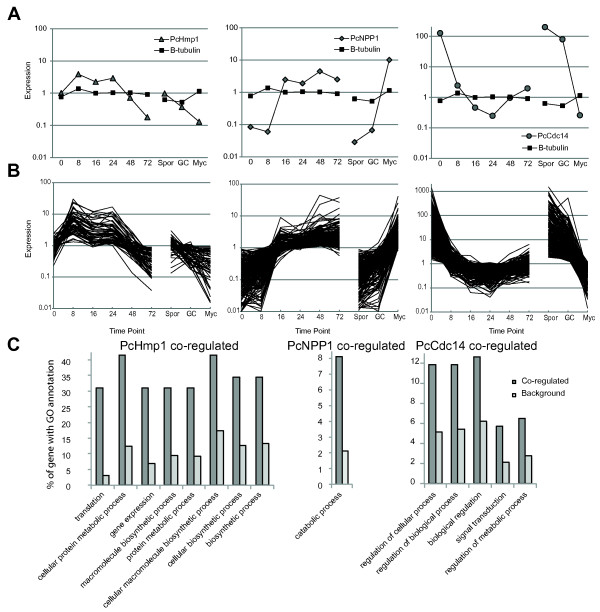
**Marker gene-assisted identification of stage-specific processes in *P. capsici***. **(A) **Expression of *PcHmp1 *(left panel), *PcNpp1 *(middle panel), and *PcCdc14 *(right panel) as determined by whole-genome microarray analyses and compared with the constitutive control gene β-tubulin. **(B) **Marker genes were used in cluster analyses to identify genes that were coregulated. The *y*-axis represents fold change in expression values, determined by calculating fold changes over mean expression values across all treatments. **(C) **Overview of significantly enriched ontologies present in marker coregulated genes. Dark bar shows the percentage of genes in the coregulated fraction compared with the background fraction (light grey). All ontologies shown were significantly enriched (*P*<0.05, false discovery rate <0.05).

To understand the infection process in more detail, we explored the *P. capsici *gene model set. We used pre-existing information to identify genes that mark specific infection stages in *Phytophthora*, and assessed their gene-expression profiles across our time-course experiment. Expression of *PcHmp1 *(*P. capsici *ortholog of the *P. infestans *Haustorial membrane protein 1, *Pi*Hmp1) [30], *PcNpp1 *(Nep1-Iike Protein 1) [31,32], and *PcCdc14 *[33], markers for biotrophy, necrotrophy, and sporulation respectively, showed distinct expression patterns in both the microarray (Figure 3A) and reverse transcription (RT)-PCR (see Additional file 11: Figure S2) data, and are consistent with stage-specific gene expression reported in other *Phytophthora *species [34]. Our results also agreed with the phenotypic changes and disease progression seen in our infection assays on tomato leaves (Figure 1A,B; see Additional file 10: Figure S1).

To refine our view of transcriptional changes associated with infectious stages in *P. capsici*, we further explored the differentially expressed gene model set and used microarray-derived expression values to identify *P. capsici *genes that are coregulated with *PcHmp1*, *PcNpp1*, and *PcCdc14 *(Figure 3B). Based on expression patterns, we were able to group 57 genes coregulated with *PcHmp1*, 209 genes with *PcNpp1*, and 533 genes coregulated with *PcCdc*14 (Figure 3B; see Additional file 2, Table S2). We then classified these coregulated genes based on available gene annotations and their corresponding proposed biological processes, and assessed enrichment for specific terms (Figure 3C; see Additional file 3, Table S3). These analyses showed that for the *PcHmp1 *coregulated genes, annotation terms were significantly enriched (*P*<0.05) for protein metabolism, (Gene Ontology (GO) number GO:0044267), gene expression (GO:0010467), and biosynthetic processes (GO:0034645) (Table S3). These results suggest activation of cellular machineries required for gene expression and translation. Transcriptional reprogramming would allow an increase in the production and processing of protein factors required for initiation and maintenance of biotrophy. Consistent with an association of *Hmp1 *with biotrophy, we also found candidate effector genes that are coregulated with *Hmp1 *(see Additional file 2, Table S2), suggesting effector-mediated dampening of host immune responses in biotrophy.

We consistently found a biotrophic phase in the first 24 hours after infection, followed by host tissue collapse and necrosis in our inoculation experiments, suggesting a distinct transition to necrotrophy. We thus determined expression of *PcNpp1*, a marker for this transition, and identified 209 genes that are coregulated in *P. capsici *(Table S2). Annotation-term enrichment analyses of this gene set showed specific enrichment for catabolic processes (GO:0009056) (Table S3). Within this gene set, a large number of peptidases and proteasomal subunits were present, suggesting active involvement of proteosomal degradation of pathogen proteins during the transition from biotrophy to necrotrophy (Table S2). Although the mechanisms of this proteasomal machinery and its targets need to be characterized, our results suggest dramatic shifts in protein modification and degradation processes, which may represent a committed step in disease development.

In the late infection stages, *Phytophthora *spp. form sporangia that emerge from necrotic tissues, a process that in *P. infestans *features upregulation of *Cdc14*. We identified *Cdc14 *coexpressed genes that were again enriched for signal transduction (GO:0007165) and metabolic processes (GO:0019222) (Table S3), which could be required for extensive cellular reprogramming underpinning spore formation. Altogether, these results are consistent with the view that *Phytophthora *infection features stage-specific transcriptional programs [34].

### *P. capsici *infection features dynamic transcriptional regulation of effector-coding genes

**Figure 4:**
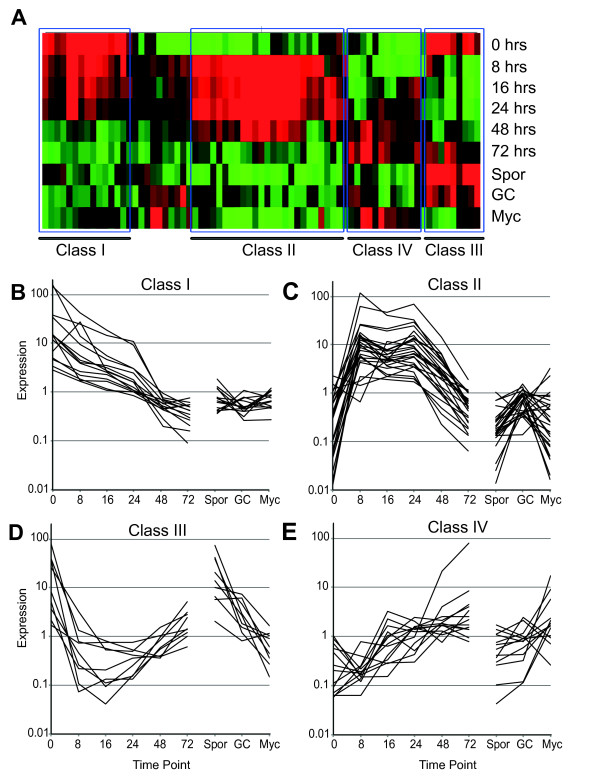
**Identification of classes of differentially expressed RXLR genes in *Phytophthora capsici***. **(A) **Cluster analyses of putative RXLR genes that were found to be differentially expressed across infectious and developmental stages identified four different groups of genes. **(B-E) **Overview of expression patterns corresponding to the groups shown in (A). Values on the *y*-axis represents fold change over mean expression as determined across all treatments.

To learn about expression of known effector genes in *P. capsici*, we extracted expression profiles for RXLR coding genes identified in the *P. capsici *genome [7] (see Additional file 4, Table S4). We detected expression of 346 RXLR-encoding genes (73%) in all tested stages and treatments, of which 73 were differentially expressed (Figure 4A; see Additional file 4, Table S4) during infection. We grouped the RXLR genes based on differential expression patterns, and defined four classes using cluster analyses (Figure 4B-E). These analyses identified 26 genes upregulated during biotrophy (8 to 24 hpi), and 13 RXLRs that were expressed in the early infection stages (0 to 16 hpi) but showed lower expression at only one biotrophic time point (24 hpi) and during necrotrophy. We also identified 9 RXLR genes that were expressed only in the sporulation stages and 13 genes that were specifically expressed in the late infection stages. In addition, 273 RXLR protein-encoding genes were expressed without any significant changes in transcript levels (see Additional file 4, Table S4). This shows that regulation of the expression of *P. capsici *genes takes place both before and during infection.

These results suggest an active involvement of pathogen effector proteins in the initiation and progression of disease, facilitated by modification of host cellular processes.

### Host transcriptional changes associated with *P. capsici *infection

**Figure 5:**
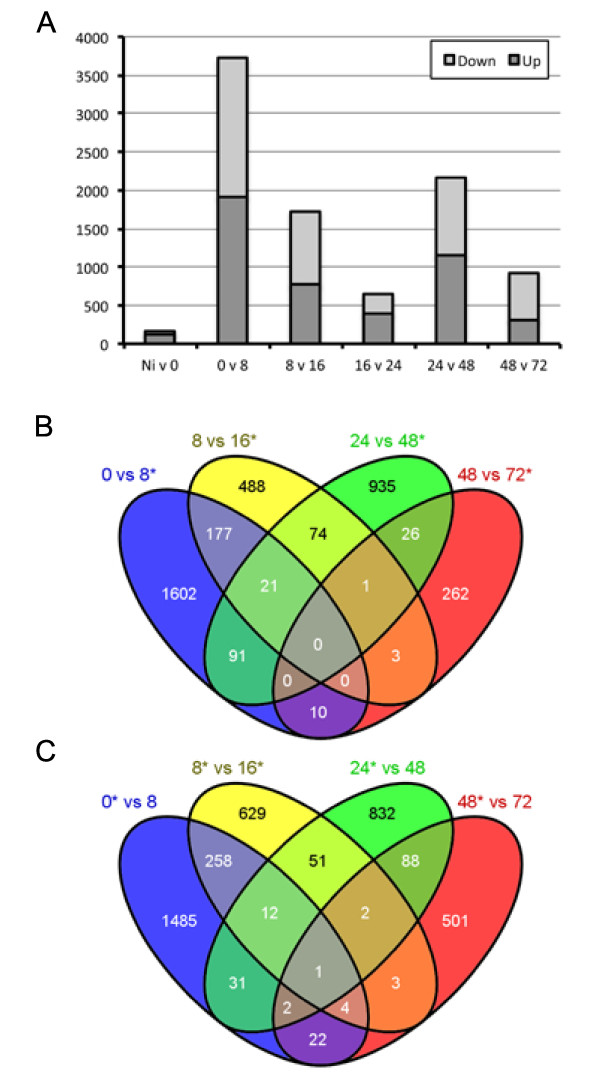
***Phytophthora capsici *infection of tomato results in two distinct transcriptional responses**. **(A) **Overview of the number of significantly upregulated (dark grey) or downregulated (light grey) between adjacent timepoints. Differences in the number of differentially expressed genes can be seen between specific early (0 versus 8 hpi) and late (24 versus 48 hpi) time-point comparisons. The non-infected (Ni) tissue was a water-inoculated control sample. Comparisons between gene lists generated in pairwise comparisons revealed limited overlap in both **(B) **upregulated and **(C) **downregulated gene sets. Diagrams were generated using Venny [49].

To learn more about *P. capsici*-mediated changes in host gene expression, we simultaneously measured host gene expression during infection with *P. capsici*. Measurements of transcript levels for 34,727 gene models (ITAG, version 2.3 [35]) revealed detectable expression of 24,390 genes at a minimum of one time point, representing 65% of the predicted tomato transcriptome. We aimed to identify genes that were differentially expressed in the time-course experiment, and identified 12,883 genes for which significant changes in gene expression were measured (one-way ANOVA, using Benjamini and Hochberg multiple testing correction, *P*≤0.005). Given that a significant change may occur between two unrelated treatments or time points (for example 8 versus 72 hpi), we also used pairwise comparisons (Student's *t*-test) to identify genes that are differentially regulated between adjacent time points (Figure 5). These analyses identified a set of 7,314 non-redundant tomato genes, suggesting dynamic transcriptional changes in tomato gene expression over the course of infection (see Additional file 5, Table S5). We determined the number of differentially expressed genes per comparison, and noted large differences in the numbers of genes that are either upregulated or downregulated between time points (Figure 5A). Our analyses suggest a major shift in gene expression (3,720 genes) between the 0 and 8 hour time points (Figure 5A; see Additional file 6, Table S6) suggesting drastic transcriptional reprogramming associated with *P. capsici *ingress and disease establishment. The water-inoculated Ni tissue was used as a control sample, and showed no significant upregulation of genes. Comparisons between the later infection stages revealed a further major shift in gene expression between the 24 and 48 hour time points (Figure 5A; see Additional file 6, Table S6), coinciding with the transition from biotrophy to necrotrophy observed during infection (Figure 1, Figure 3).

Given the dramatic changes in gene expression, we determined the level of overlap of differentially expressed genes between sampled time points (Figure 5B,C). These analyses revealed only a limited number of genes that were upregulated or downregulated at multiple time points, and large suites of genes that were uniquely regulated between 0 versus 8 (2,087), 8 versus 16 (1,117), and 24 versus 48 (1,757) hpi (Figure 5B,C). Crucially, little overlap was found between differentially expressed gene sets from the 0 versus 8 hpi and 24 versus 48 hpi comparisons (Figure 5B,C). These results suggest two major but distinct transcriptome changes in the host occurring at initial infection (0 to 8 hpi) and the transition from biotrophy to necrotrophy (24 to 48 hpi).

### *P. capsici *infection leads to two distinct transcriptional responses in tomato

**Figure 6:**
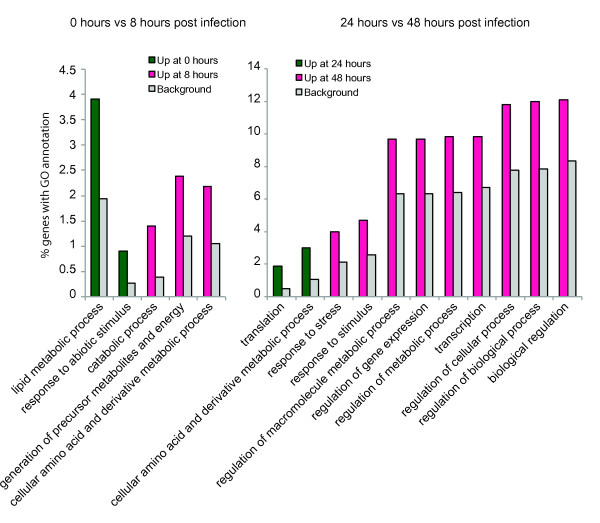
**Gene ontology (GO) enrichment analyses of tomato genes identified in the early (0 versus 8) and late (24 versus 48) transcriptional response**. Percentage of genes with significantly enriched GO terms that were specifically expressed in either of the time points (magenta/green) in our pairwise comparisons, compared with the background (grey). The *y*-axis shows the percentage of genes falling within each given GO annotation class.

To identify the biological processes affected by those two distinct responses, we assessed relative enrichment of annotation categories from genes that were present in both our ANOVA and pairwise comparisons sets. As expected, we found no enrichment of processes in the non-infected versus 0 hpi time points, partly owing to a relatively small number (*n *= 171) of genes that were differentially expressed between these treatments. However, further assessment of sets emerging from other comparisons revealed significant enrichment for specific processes at the 0 versus 8 hpi and 24 versus 48 hpi time points (Figure 6; see Additional file 7, Table S7). Processes associated with (primary) metabolism (GO:0008152, GO:0044238) were significantly enriched in the fraction upregulated at 0 hpi, suggesting a drop in core metabolic genes after infection (Figure 6; see Additional file 7, Table S7). Both catabolic processes (GO:0009056) and specific metabolic processes were enriched in the fraction upregulated at 8 hpi, suggesting major metabolic reprogramming in early infection (Figure 6,; see Additional file 7, Table S7).

The switch from 24 to 48 hpi showed drastic re-regulation of metabolic and biosynthetic processes. Interestingly, the genes specifically upregulated at 48 hpi showed enrichment for a relatively large number of ontologies, including response to stimulus (GO:0006950) and response to stress (GO:0050896), and a number of gene regulation-related ontologies (Figure 6; see Additional file 7, Table S7). These results suggest an active response of the host that accompanies the initiation of necrotrophy by *P. capsici*, suggesting a pathogen-derived cue that causes host cell death. If true, perturbation of this process may limit initiation of pathogen necrotrophy, which in turn could lead to reduced pathogen growth and sporulation.

### *P. capsici *infection features differential expression of candidate PAMP perception and signaling genes in tomato

We noted a vast transcriptional shift in tomato between the 0 and 8 hpi time points, and hypothesized that these changes are due either to an initial PAMP or effector induced-response upon pathogen ingress. We also hypothesized that upon infection, the PTI response is dampened by effectors that are expressed and delivered during infection and biotrophy (Figure 4). If true, immune signaling gene candidates that help determine interaction outcomes could be identified. We thus investigated transcriptional changes in gene classes involved in pathogen perception and signaling.

**Figure 7:**
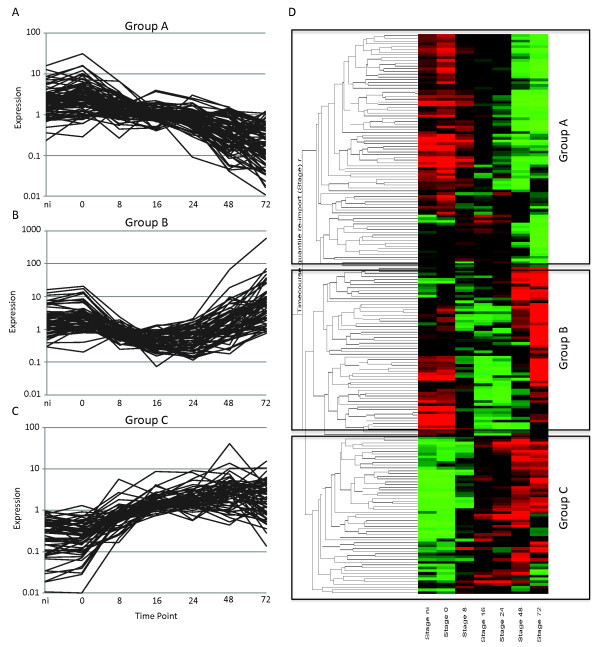
**Differentially expressed immune signaling candidate genes identified in microarray analyses**. Overview of differentially expressed immune signaling candidate genes identified in pairwise comparisons between time points (Student's *t*-test) and ANOVA (*P *= 0.005) analyses. **(A-C) **Expression profiles are presented for class A, B, and C genes, identified by **(D) **cluster analyses in Genespring. Red and green represent upregulated and downregulated genes respectively. The *y*-axis shows average linkage of Pearson correlations of gene-expression profiles. The non-infected (Ni) tissue was a water-inoculated control sample.

We exploited available annotations for tomato gene models in MAPMAN, and identified 202 signaling genes in our differentially regulated dataset (Figure 7; see Additional file 8, Table S8). Subsequent cluster analyses and classification revealed a set of 84 genes that are induced between 0 and 8 hpi but that decrease in expression in the later stages (group A) (see Additional file 8, Table S8) and 61 genes that appear to be specifically suppressed in biotrophy (group B) (see Additional file 8, Table S8). Another group of 57 genes (group C) (see Additional file 8, Table S8) were found to be transcriptionally activated throughout the time course after pathogen ingress, possibly reflecting activation of signaling networks that allow pathogen growth. Notably, in our differentially regulated set, we identified large suites of receptor-like kinases (RLKs) that are downregulated in biotrophy (group B), suggesting that they may be targeted by *P. capsici *effectors and their downregulation may enhance virulence. We noted that a large proportion of differentially regulated receptor-like genes are annotated as receptors involved in nodulation, suggesting overlap between symbiotic and pathogenic associations with host plants. Importantly, in our set of RLKs, we identified a homolog of AtPepR1 [36] that is suppressed during disease progression (group B), suggesting that immune suppression is important in biotrophy. Approaches that aim to maintain or enhance their expression during biotrophy may limit disease progression.

### Differential regulation of host transcription factors underpins transcriptional responses to *P. capsici *infection in tomato

**Figure 8:**
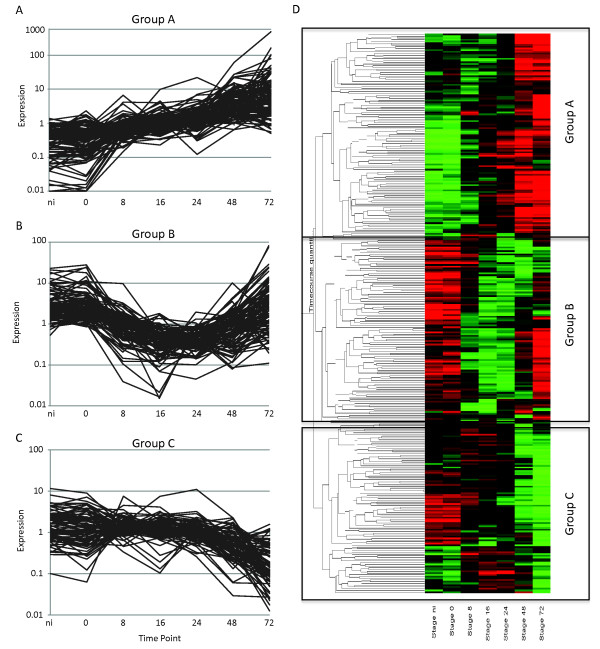
***Phytophthora capsici *infection leads to dynamic changes in host transcription-factor genes**. Overview of differentially expressed candidate transcription-factor genes, identified in pairwise comparisons between time points (Student's *t*-test) and ANOVA (*P *= 0.005) analyses. **(A-C) **Expression profiles are presented for class A, B, and C genes, identified by **(D) **cluster analyses in Genespring, showing distinct expression changes during infection. Red and green represent upregulated and downregulated genes respectively. The *y*-axis shows average linkage of Pearson correlations of gene-expression profiles. The non-infected (Ni) tissue was a water-inoculated control sample.

The observed dramatic changes in gene expression, both early in infection and during the transition from biotrophy to necrotrophy, prompted us to extract expression profiles for known and differentially regulated transcription factors (Figure 8). Clustering and subsequent grouping of these transcription factors based on expression patterns revealed the presence of distinct transcriptional profiles, consistent with wholesale transcriptional changes in tomato during infection. A large group of transcription factors was found to be induced upon infection (class A) (Figure 8; see Additional file 8, Table S8), whereas others were either repressed during biotrophy (class B) (Figure 8; see Additional file 8, Table S8) or were expressed throughout infection but specifically downregulated during necrotrophy (class C) (Figure 8; see Additional file 8, Table S8). We assessed transcription-factor family membership for each of these expression classes, and found that class A contained a relatively large fraction of the WRKY-type transcription-factor families (see Additional file 8, Table S8).

These results are consistent with the activation of genes involved in (biotic) stress responses, and suggest execution of specific transcriptional programs possibly leading to tissue necrosis. Our results also suggest involvement of the phytohormone ethylene and its responsive transcription factors in disease development, as a sizeable fraction was found to fall into classes A and B (Figure 8; see Additional file 8, Table S8). These results suggest repression of specific transcriptional regulators by *P. capsici *during early and biotrophic infection stages. Taken together, our results lead us to suggest that further investigation into and alteration of specific transcriptional changes leading to necrosis in hosts may prevent or limit progression of *P. capsici *infection beyond biotrophy and limit sporulation.

## Discussion

### A composite whole-genome microarray approach to study plant-microbe interactions in new detail

In this paper, we report on a genome-wide analysis of transcriptional changes that take place in tomato and its pathogen *P. capsici*. By utilizing their full genomes, our work provides the first detailed simultaneous overview of gene-expression changes during the course of infection in both a pathogen and its plant host. This approach allows an unprecedented view in great detail of the processes that underpin infection, disease progression, and lifestyle transitions. Given the immense damage *Phytophthora *species continue to cause in important crops, these analyses will thus provide new means and exciting opportunities to investigate complex yet important plant-microbe interactions, in which extensive signal interplay is known to occur. Our approach sets the stage for standardized experiments that can compare the effect of pathogen infection strategies on a given host, or the importance of host factors on pathogen transcriptional programs.

### *P. capsici *infection features a hemi-biotrophic lifecycle

Using confocal microscopy and microarray analyses, we found evidence of a distinct biotrophic phase, followed by transition to necrotrophy after 24 hpi and sporulation at 72 hpi on susceptible tomato (Figure 1). Biotrophy is marked by the formation of distinct haustorial structures that invaginate living cells, an important feature we were able to show on *N. benthamiana *plants expressing ER-eGFP (see Additional file 10: Figure S1). These results are similar to observations made in other *Phytophthora *species [30,37,38], although we note that *P. capsici *has a relatively short infection cycle compared with related organisms such as *P. infestans *[27]. We took advantage of the availability of genome sequences for both *P. capsici *and tomato, and used this information to design a custom two-genome array and measure gene expression in both organisms in a detailed time-course experiment. Using this approach, we were able to measure and demonstrate the expression of 20,530 *P. capsici *and 24,390 tomato genes in a replicated time-course experiment. We determined transcriptional programs associated with distinct stages of pathogen infection.

### The *P. capsici *lifecycle is marked by activation of stage-specific processes

Using the existing literature, we identified and selected three *Phytophthora *marker genes that could provide information on disease progression and development after infection. Assessment of expression for *PcHmp1*, *PcNpp1*, and *PcCdc14 *during *P. capsici *infection confirmed the presence of a hemi-biotrophic lifecyle that features biotrophy in the first 24 hpi, a switch to necrotrophy between 24 and 48 hpi, and sporulation at 72 hpi (Figure 2). Identification of coregulated genes followed by GO term enrichment analyses revealed that genes associated with expression and translation of genes and with protein metabolism were over-represented in biotrophy (Figure 3). These analyses suggest that the *de novo *expression, production and modification of proteins are crucial requirements for initiation and maintenance of biotrophy, and that *P. capsici *does not take amino acids directly from the host. This is consistent with previous work showing that haustoriating *P. infestans *cysts show relatively high expression of amino acid biosynthesis genes [39].

Given that the plant-haustorial membrane interface is a crucial site where effector proteins are secreted and delivered into host tissues and cells, it is plausible that *Hmp1*-coregulated genes are required for haustorial development and enhancement of effector protein production and delivery. The identification of stage-specific genes, encoding secreted proteins of unknown function or cellular destination, may help identify novel effector (classes) and help determine their roles in virulence.

The NEP1-like protein superfamily forms an important class of necrosis-inducing peptides with proposed roles in pathogen virulence. In this study, we found that, together with *PcNPP1*, a significant group of genes is induced during the transition from biotrophy to necrotrophy, suggesting a committed transcriptional shift between stages. Enrichment analyses revealed a significant gene complement associated with catabolism and degradation, suggesting that transcriptional shifts may result in cellular reprogramming of *Phytophthora *hyphae or accommodate the breakdown of compounds released during host cell death. These results illustrate a dynamic transcriptional program used by *P. capsici *to drive differentiation and adaptation.

Successful *Phytophthora *infection must lead to the formation of sporangia, an essential developmental process required for propagation of the *Phytophthora *disease cycle. Given the devastation caused by often explosive *Phytophthora *epidemics, there is considerable interest in the mechanisms governing sporulation and dissemination. In this study, we assessed genes coregulated with the sporulation marker *Cdc14 *and identified genes required for signaling, regulation, and expression. These results are consistent with the idea of extensive signaling cascades that drive the formation and differentiation of sporangia from a hyphal stage. Although the exact cascades driving sporulation still require elucidation, our work, together with gene-expression studies on other *Phytophthora *spp., should allow identification of common genes associated with spore formation, which in turn could inform strategies that stop pathogen dissemination and limit epidemics on crops. Interestingly, a relatively small percentage (22%) of differentially expressed genes fall into one of the coregulated gene sets. Although this could be due to high levels of stringency during our analyses, it could also point to the existence of other coregulated gene classes, driving as yet unknown processes required for disease development. More detailed cluster analyses and investigation of candidate gene function may thus give rise to additional sets of marker genes, suited to study *Phytophthora *infection.

### *P. capsici *effector gene expression is regulated by developmental and plant signaling cues

We assessed gene-expression patterns for the RXLR class of effectors, and detected expression for a relatively high proportion of RXLR-coding genes (73%). These results could be due to the high level of sensitivity provided by the Agilent platform (as evidenced by the large number of *P. capsici *genes detected at 0 hpi) or the number of time points and stages assayed in our microarray experiments (9), or might reflect the biology of a pathogen that has a broad host range. We found that, based on expression changes, RXLR genes can be grouped into four distinct classes. Class I and III RXLRs were highly expressed in the early phase of infection, and showed either low (class I) or high (class III) expression levels in germinating cysts. These results suggest the presence of both a developmental program and specific plant signals that drive RXLR gene induction. Given their expression early in infection, these genes are likely to play roles in prevention or suppression of initial immune responses. Besides genes expressed in the early infection stages, we also found RXLR-coding genes that were upregulated in biotrophy (classes II and IV), most of which were downregulated in necrotrophy (class II), and some that remained highly expressed in the late stages (class III). Given the observation that biotrophy features suppression of defense responses, we suggest that secretion and delivery of effectors are required for the maintenance of biotrophy. Our results also suggest continuous reprogramming of host cells in favor of pathogen growth. Effector genes expressed in the late stages could stimulate cell death in the necrotrophic phase or modulate host metabolism.

### *P. capsici *infection and disease progression induces two distinct responses in tomato

By characterizing host gene expression during *P. capsici *infection, we identified processes associated with pathogen infection and lifestyle. Pairwise comparisons between time points identified two distinct transcriptional changes in tomato, coinciding with initial infection (0 versus 8 hpi) and the transition from biotrophy to necrotrophy (24 versus 48 hpi). Characterization of the early response revealed downregulation of genes required for primary metabolism, whereas genes falling into secondary metabolism categories were induced as part of early responses to infection. These results are in line with previously reported observations, and could reflect production of antimicrobial compounds upon initial *Phytophthora *ingress.

### *Phytophthora *infection results in the induction and suppression of PAMP-like responses

Whereas some *Phytophthora*-host interactions feature suppression of initial host defense responses [40], we found evidence suggesting defense responses occurring early in infection. These included differential regulation of genes encoding RLKs, including the PEPR1 receptor and classes with similarity to Nod factor receptors. These results suggest activation of PAMP or effector-triggered immune responses that may overlap with pathways that are regulated by Nod receptor-like genes in plants. These results may indicate co-opting of signaling pathways normally activated in symbiosis, and would give weight to recent observations made in *Lotus japonicus *[41]. Crucially, in a set of differentially expressed RLK-coding genes, we identified a subset of candidate receptors whose expression was specifically repressed in biotrophy. These results, together with the identification of effectors induced in the early stages of infection, lead us to suggest that, consistent with current models describing plant-microbe interactions, *P. capsici *secretes and delivers effectors into host tissues to limit PAMP perception, inhibit immune signaling, and promote virulence. With both effector and immune signaling genes now characterized in *P. capsici *and tomato respectively, it is now possible to investigate the mechanisms driving *P. capsici *virulence and host immune signaling. Over-expression of host RLKs normally downregulated in biotrophy may lead to enhanced PTI responses that limit pathogen growth and disease development.

## Conclusions

Our results suggest dynamic but highly regulated transcriptional programming in both host and pathogen that underpin *P. capsici *disease and hemi-biotrophy. We found expression changes in both effector-coding genes and host factors involved in immunity, suggesting distinct roles for effectors towards susceptibility by modulating host processes. With new unprecedented detail on transcriptional reprogramming during infection in both host and pathogen, the coordinate relationships that drive host-microbe interactions and the basic processes that drive hemi-biotrophy can now be explored. Importantly, and with the availability of genome sequences for both hosts and distinct classes of pathogens that share parasitic lifestyles, it is possible to identify and study the processes that underpin pathogen lifestyles. Given that major transcriptional switches can be observed in both *Phytophthora *and tomato during infection, deliberate alteration of lifestyle-associated transcriptional changes may allow prevention or perhaps disruption of hemi-biotrophic disease cycles, and limit damage caused by epidemics.

## Methods

### Plant material

*S. lycopersicum *'Moneymaker' plants were grown in controlled growth chambers at 22°C, with a photoperiod of 16 hours, supplemented by artificial light. The third leaf from the top of every plant was detached and placed upside-down in humid transparent plastic trays in a controlled incubator with the same settings as in the growth chamber. Leaf discs centered on mock-inoculated tissue and infected lesion tissue were harvested using a cork borer (diameter 7 mm), and frozen in liquid nitrogen before RNA extraction.

### *P. capsici *inoculation and *in vitro *samples

*P. capsici *wild-type strain LT1534 was grown in petri dishes on V8 agar medium in a dark climate chamber at 25°C for 4 days and under standard light at 22°C for 3 days. To induce zoospore release, plates were flooded with ice-cold distilled water, and spores were harvested from sporulating mycelia by dislodging the sporangia with a sterile glass spreader. Sporangial suspensions were collected and incubated at room temperature under bright light conditions. Release of zoospores was monitored, and their numbers counted in a hemocytometer under a microscope, and adjusted to 1 × 10^5^/ml. The detached leaves were inoculated with four 20 µl droplets of the zoospore solution. In addition to samples taken during the infectious stages, three *in vitro *samples were taken: sporangia/zoospores (Spor), germinating cysts (GC), and mycelia (Myc) grown *in vitro*. Spor (taken at 0 hpi) and GC (taken at 16 hpi) were sampled from the same inoculum/sporangial suspension, differing only in harvesting times. They were collected from 10 ml of sporangial suspension after an incubation time of 1 h (Spor) and 16 h (GC) at 22 °C. The mycelia were grown in 1 ml pea broth, infected with 20 µl of inoculum at 22°C, and harvested 48 hpi by collecting the mycelial mat into 10 ml tubes. All samples were placed in the controlled incubator with the same settings and conditions as the leaf samples, and harvested after centrifugation for 2 minutes at 1,200 × g. After the supernatant was removed, the pellets were collected and frozen in liquid nitrogen.

### RNA extractions and cDNA synthesis

RNA was isolated from frozen leaf tissue (RNeasy Plant Mini Kit; Qiagen Inc., Valencia, CA, USA) and treated afterwards with DNAse (Ambion, Foster City, CA, USA) to remove genomic DNA contamination, in accordance with the instructions of the manufacturers. To test for genomic DNA contamination, PCR using primers specific for *PcTubulin *(Table 1), was performed on the extracted RNA. cDNA was synthesized using 500 ng of total RNA, using a commercial kit and primer (SuperScript™ II cDNA synthesis kit and Oligo dT primer; Invitrogen Corp., Carlsbad, CA, USA) following the manufacturer's instructions.

**Table 1:** Primers used in study.

Primer	Direction	Sequence
*PcTubulin*	Forward	GACTCGGTGCTTGATGTTGTC
	
	Reverse	CCATCTCATCCATACCCTCGCCAG

PcHmp1-F	Forward	CATGATGGCAGTCATGGTCGGTGAAG

*PcHmp1*-R	Reverse	TTAGCTAACATTGAGGCGGGCATGCAG

*PcNPP1*-F:	Forward	CAGCTCCACATCACCAACGGct

*PcNPP1*-R	Reverse	CTCTTCCCGTTCAAATAGTTC

*PcCDC14*-F	Forward	GGAAGCGATTGAGTTCTTGC

*PcCDC14*-R	Reverse	TTCTCCACACGCTCAAAGTG

### Microarray design and analysis

A custom 60-mer oligonucleotide microarray was designed from predicted transcripts of the *P. capsici *(LT1534 v11.0 [7]), and *S. lycopersicum *(ITAG 2.3, [35]) genomes using eArray software (Agilent Technologies, Inc., Santa Clara, CA, USA). The *P. capsici *predicted transcriptome (Phyca11_No.) was supplemented with separately predicted CRN effectors (Scaffold_No.) as described by Stam *et al. *[23], and RXLR effectors (PhycaSCAFFOLD_No.) as described below. The design and sequences are available at ArrayExpress (accession A-MEXP-2253), and represent 20,530 transcripts for *P. capsici *and 34,510 transcripts for *S. lycopersicum*. RNA labeling and microarray hybridization procedures were performed (Genome Technology, The James Hutton Institute, Dundee, UK) as described previously. In short, fluorescent one-color labeling of the RNA and hybridization was performed as recommended (Agilent One-Color Microarray-Based Gene Expression Analysis (Low Input Quick Amp Labeling) version 6.5; Agilent Technologies, Inc., Santa Clara, CA, USA) using 8 × 60 k format slides.

The microarray experimental design, along with raw datasets, is available at ArrayExpress (Accession A-MEXP-2253). The extracted dataset was separated for each array into *P. capsici *and *S. lycopersicum *data to allow independent processing of each dataset. Datasets were each independently quality filtered using flag values (present or marginal in two-thirds of replicates) and then quantile-normalized with Genomics Suite software (Partek Inc,. St Louis, MO, USA), before being loaded into Genespring (version 7.3; Agilent Technologies) software for analysis. Statistical tests were performed using one-way ANOVA (Benjamini and Hochberg multiple testing correction, *P*≤0.005) to identify significantly changed genes across the *in planta *time course. For grouping genes that are coregulated with markers of *Phytophthora *infection stages, a minimum Pearson correlation of at least 85% was used to define clusters. Candidate secreted proteins were identified by using SignalP (version 3) analyses on the predicted *P. capsici *proteome [42], applying a hidden Markov model (HMM) cut-off score of less than 0.5. Predicted membrane proteins were identified using TMHMM [43], and removed from the secreted protein set as described previously [23]. This set was then augmented with predicted RXLRs (this study) and previously described CRN gene models [23].

### Marker gene sequences

For all marker genes, the original *P. infestans *sequences were retrieved from NCBI using the published accession numbers (*PiHmp1: *EU680858.1; *PiNPP1*: AF356840.1; *PiCDC14*: AY204881.1). The sequences were then used in a tBLASTn and BLASTp [44] search against the *P. capsici *genome version 11 [7] to obtain the corresponding *P. capsici *homologous sequences, for which primer pairs (Table 1) were designed. Reverse transcription PCR was performed in 25-µl reaction volumes with 1 µl of cDNA (1:5 dilution) as template. Thermocycling conditions of the PCR were: 94°C for 2 minutes, followed by 30 cycles at 94°C for 30 seconds, 57 C for 30 seconds and 72°C for 2.5 minutes. Extension was finalized at 72°C for 10 minutes to allow trimming of incomplete polymerizations. Amplicons from cDNA were Sanger-sequenced, and derived sequences were aligned to *P. infestans *reference sequences using the program ClustalW Multiple Sequence Alignment [45] to investigate the levels of sequence similarity. *P. capsici *marker genes were highly similar to those identified in *P. infestans*, with the similarity of the protein sequences being 77% for HMP1 and NPP1, and 88% for CDC14. [We refer here to proteins]

### Identification of PcRXLR complement

Analysis of previously published *P. capsici *RXLRs [7] indicated that the present database was incomplete. We therefore implemented a new identification strategy, in which RXLRs were sought using previously published methods [13,46,47]. All output was collated and compared with previously predicted *P. capsici *RXLR component using BLASTN. Redundancies were removed, and in cases of difference in predicted open reading frame length, sequences were compared with known PiRXLR sequences and manually curated. This yielded a set of 516 RXLR candidates, of which 471 were represented on the array (see Additional file 9, Table S9).

### Confocal imaging

Zoospores (5 × 10^5^/ml, generated as described above) of transformed *P. capsici *LT1534:tdTomato were inoculated in 20 µl droplets onto leaves of *S. lycopersicum *'Moneymaker' or *N. benthamiana *(Line 16c) plants. Plants were incubated in a small climate chamber to maintain humidity and kept at 20°C for a maximum of 72 hours to allow *P. capsici *to infect leaves, form haustoria, and colonize host tissues. Imaging was conducted on a confocal microscope (LSM 710; Zeiss, Jena, Germany) using a water dipping lens (W Plan-Apochromat 40x/1.0 DIC M27; Zeiss) and the following settings: tdTomato (561 nm excitation and 573 to 612 nm emission) and chlorophyll (488 nm excitation and 650 to 700 nm emission). Haustoria are indicated with white arrows. The scale bars shown are 20 μm.

### GO enrichment analysis

To investigate enrichment of specific gene ontologies in either *P. capsici *marker coregulated genes or *S. lycopesicum *genes in our pairwise analysis, we used a singular enrichment analysis (SEA) strategy. All genes with no GO annotations were filtered from the set, and compared with a customized background set containing all genes on the array with known ontologies for *P. capsici *or tomato, respectively. SEA was done using AgriGO. Significance was tested using Fisher's exact test, results were reported for *P*<0.05 after correction for false discovery rate [48]. *P. capsici *results were reported using GO Slim annotations, and tomato results with GO plant Slim.

## Authors' contributions

JJ carried out time-course experiments and microarray sample preparation, and JJ, PH, RS, and EH designed the custom array. The technical part of the microarray was carried out by JM and JJ. Quantile normalization was performed by RZ and PH. *P. capsici *analysis was carried out by JJ, PH and RS and tomato analysis by RS, EH, and PH. Confocal microscopy was performed by AH. EH, JJ, RS, and AH wrote the manuscript. All authors read and approved this manuscript.

## Supplementary Material

Additional file 1 **Table S1**. *Phytophthora capsici *genes encoding putative secreted proteins found expressed in microarray experiments. Lists of *P. capsici *genes encoding candidate secreted proteins are given. These represents gene for which expression was detected in the infectious and *in vitro *stages. Click here for file

Additional file 2 **Table S2**. *PcHmp1*, *PcNPP1*, and *PcCdc14 *coregulated genes Overview of genes found to be coregulated with marker genes in *Phytophthora capsici*. Gene identifier, probe ID number, and normalized expression value (fold change over mean expression) are given for each gene and at each time point. Gene Ontology (GO) annotations are also given for each gene where available. Click here for file

Additional file 3 **Table S3**. Significantly enriched ontologies coregulated with marker genes, Overview of gene ontologies that are significantly enriched in the fractions that are specifically coregulated with *P. capsici *marker genes as shown in Figure 3C. Gene Ontology (GO) terms, *P*-values, false discovery rates (FDRs), and query and reference sample sizes are given. Click here for file

Additional file 4 **Table S4**. *Phytophthora capsici *candidate RXLR genes expressed in microarray experiments. Overview of RXLR effector genes found to be upregulated during specific lifecycle stages in *P. capsici*. Gene identifier, probe ID number, and normalized expression value (fold change over mean expression) are given for each gene and at each time point. Genes are listed per class as shown in Figure 4. Click here for file

Additional file 5 **Table S5**. Annotation and expression of tomato genes differentially expressed in pairwise comparisons. Overview of genes found to be differentially expressed between timepoints during *Phytophthora capsici *infection. Gene identifiers, normalized expression values, and Gene Ontology (GO) annotations are given. Click here for file

Additional file 6 **Table S6**. Tomato genes differentially expressed in each pairwise comparison. Overview of genes found to be differentially expressed during *Phytophthora capsici *infection in pairwise comparisons between two timepoints. Gene identifiers and normalized expression values are given. Click here for file

Additional file 7 **Table S7**. Genes corresponding to enriched gene ontologies (GOs) in pairwise comparisons. Overview of GOs that were significantly enriched in the fractions that were specifically upregulated or downregulated between two time points as shown in Figure 6. GOs, *P*-values, false discovery rates (FDRs), and query and reference sample sizes are given. Click here for file

Additional file 8 **Table S8**. Tomato genes with possible roles in pathogen-associated molecular pattern (PAMP) perception and signaling, differentially expressed during *Phytophthora capsici *infection. Overview of candidate PAMP signaling and transcription-factor genes found to be differentially expressed during *P. capsici *infection. Gene identifiers, normalized expression values, and annotations are given. Genes are grouped based on their expression patterns as shown in Figure 7 and 8 respectively. Click here for file

Additional file 9 **Table S9**. Putative RXLR effectors used in this study. Overview of putative RXLR effectors. Gene name, probe ID, and nucleotide sequence are given for each gene. Click here for file

Additional file 10 **Figure S1**. Assessment of cell viability during *Phytophthora capsici *infection. Transgenic *Nicotiana benthamiana *plants constitutively expressing ER-eGFP, a green fluorescent protein (GFP) localized to the endoplasmic reticulum (ER) were used to assess whether host cells were alive during the course of infection. **(A) **Photographs of *N. benthamiana *leaves infected with zoospore suspensions of *P. capsici *at 0, 8, 24, 48, and 72 hpi. **(B) **Confocal microscopy images of *N. benthamiana *leaves infected with a transgenic *P. capsici *strain expressing the fluorescent protein TdTomato. Within the first 24 hours, the host ER was largely intact despite the presence of *P. capsici*, and haustoria were often seen to invaginate living cells. After 24 hours, the ER network was disrupted as shown by the unstructured distribution of GFP, suggesting dead or dying cells. Bar = 20 μm. Click here for file

Additional file 11 **Figure S2**. Reverse transcription PCR verification of marker-gene expression during infection. Expression of the marker genes *PcHmp1*, *PcNpp1*, *PcCdc14*, and *PcTub *(constitutive control) was tested by semi-quantitative PCR on cDNA derived from a time-course infection series used for the microarrays. Amplification of genes on cDNA derived from water-inoculated control (non-infected; ni) and tomato harvested 0, 8, 16, 24, 48, and 72 hpi with *Phytophthora capsici*. Click here for file

## References

[B1] Jones JDG Dangl JL The plant immune system. Nature 2006 14 323 329 10.1038/nature05286 17108957

[B2] Birch PRJ Rehmany AP Pritchard L Kamoun S Beynon JL Trafficking arms: oomycete effectors enter host plant cells. Trends Microbiol 2006 14 8 11 10.1016/j.tim.2005.11.007 16356717

[B3] Chisholm ST Coaker G Day B Staskawicz BJ Host-microbe interactions: shaping the evolution of the plant immune response. Cell 2006 14 803 814 10.1016/j.cell.2006.02.008 16497589

[B4] Kamoun S A catalogue of the effector secretome of plant pathogenic oomycetes. Annu Rev Phytopathol 2006 14 41 60 10.1146/annurev.phyto.44.070505.143436 16448329

[B5] Fisher MC Henk DA Briggs CJ Brownstein JS Madoff LC McCraw SL Gurr SJ Emerging fungal threats to animal, plant and ecosystem health. Nature 2012 14 186 194 10.1038/nature10947 22498624 PMC3821985

[B6] Lamour KH Stam R Jupe J Huitema E The oomycete broad-host-range pathogen Phytophthora capsici. Mol Plant Pathol 2012 14 329 337 10.1111/j.1364-3703.2011.00754.x 22013895 PMC6638677

[B7] Lamour KH Mudge J Gobena D Hurtado-Gonzales OP Schmutz J Kuo A Miller NA Rice BJ Raffaele S Cano L Bharti AK Donahoo RS Finley SL Huitema E Hulvey J Platt D Salamov A Savidor A Sharma R Stam R Storey D Thines M Win J Haas B Dinwiddie D Jenkins J Knight J Affourtit J Han CS Chertkov O Genome sequencing and mapping reveal loss of heterozygosity as a mechanism for rapid adaptation in the vegetable pathogen Phytophthora capsici. Mol Plant Microbe Interact 2012 14 10 1350 1360 10.1094/MPMI-02-12-0028-R 22712506 PMC3551261

[B8] Judelson HS Blanco FA The spores of *Phytophthora*: weapons of the plant destroyer. Nat Rev Microbiol 2005 14 47 58 10.1038/nrmicro1064 15608699

[B9] Horbach R Navarro-Quesada AR Knogge W Deising HB When and how to kill a plant cell: infection strategies of plant pathogenic fungi. Journal Plant Physiol 2011 14 51 62 10.1016/j.jplph.2010.06.014 20674079

[B10] Koeck M Hardham AR Dodds PN The role of effectors of biotrophic and hemibiotrophic fungi in infection. Cell Microbiol 2011 14 1849 1857 10.1111/j.1462-5822.2011.01665.x 21848815 PMC3218205

[B11] Infection structures of biotrophic and hemibiotrophic fungal plant pathogens. Mol Plant Pathol 2001 14 101 108 10.1046/j.1364-3703.2001.00055.x 20572997

[B12] van West P Shepherd SJ Walker CA Li S Appiah AA Grenville-Briggs LJ Govers F Gow NAR Internuclear gene silencing in Phytophthora infestans is established through chromatin remodelling. Microbiology 2008 14 1482 1490 10.1099/mic.0.2007/015545-0 18451057

[B13] Whisson SC Boevink PC Moleleki L Avrova AO Morales JG Gilroy EM Armstrong MR Grouffaud S van West P Chapman S Hein I Toth IK Pritchard L Birch PR A translocation signal for delivery of oomycete effector proteins into host plant cells. Nature 2007 14 115 118 10.1038/nature06203 17914356

[B14] Haas BJ Kamoun S Zody MC Jiang RHY Handsaker RE Cano LM Grabherr M Kodira CD Raffaele S Torto-Alalibo T Bozkurt TO Ah-Fong AMV Alvarado L Anderson VL Armstrong MR Avrova A Baxter L Beynon J Boevink PC Bollmann SR Bos JIB Bulone V Cai G Cakir C Carrington JC Chawner M Conti L Costanzo S Ewan R Fahlgren N Genome sequence and analysis of the Irish potato famine pathogen *Phytophthora infestans*. Nature 2009 14 393 398 10.1038/nature08358 19741609

[B15] Tyler BM Tripathy S Zhang X Dehal P Jiang RH Aerts A Arredondo FD Baxter L Bensasson D Beynon JL Chapman J Damasceno CM Dorrance AE Dou D Dickerman AW Dubchak IL Garbelotto M Gijzen M Gordon SG Govers F Grunwald NJ Huang W Ivors KL Jones RW Kamoun S Krampis K Lamour KH Lee MK McDonald WH Medina M *Phytophthora *genome sequences uncover evolutionary origins and mechanisms of pathogenesis. Science 2006 14 1261 1266 10.1126/science.1128796 16946064

[B16] Bozkurt TO Schornack S Win J Shindo T Ilyas M Oliva R Cano LM Jones AME Huitema E van der Hoorn RAL Kamoun S Phytophthora infestans effector AVRblb2 prevents secretion of a plant immune protease at the haustorial interface. Proc Natl Acad Sci USA 2011 14 20832 20837 10.1073/pnas.1112708109 22143776 PMC3251060

[B17] Saunders DGO Win J Cano LM Szabo LJ Kamoun S Raffaele S Using hierarchical clustering of secreted protein families to classify and rank candidate effectors of rust fungi. PLoS ONE 2012 14 e29847 10.1371/journal.pone.0029847 22238666 PMC3253089

[B18] Bozkurt TO Schornack S Banfield MJ Kamoun S Oomycetes, effectors, and all that jazz. Current Opinion in Plant Biology 2012 14 9 10.1016/j.pbi.2012.03.008 22483402

[B19] Oliva R Win J Raffaele S Boutemy L Bozkurt TO Chaparro-Garcia A Segretin ME Stam R Schornack S Cano LM Damme Mv Huitema E Thines M Banfield MJ Kamoun S Recent developments in effector biology of filamentous plant pathogens. Cell Microbiol 2010 14 705 715 10.1111/j.1462-5822.2010.01471.x 20374248

[B20] Birch PR Boevink PC Gilroy EM Hein I Pritchard L Whisson SC Oomycete RXLR effectors: delivery, functional redundancy and durable disease resistance. Curr Opin Plant Biol 2008 14 373 379 10.1016/j.pbi.2008.04.005 18511334

[B21] Morgan W Kamoun S RXLR effectors of plant pathogenic oomycetes. Curr Opin Microbiol 2007 14 332 338 10.1016/j.mib.2007.04.005 17707688

[B22] Schornack S van Damme M Bozkurt TO Cano LM Smoker M Thines M Gaulin E Kamoun S Huitema E Ancient class of translocated oomycete effectors targets the host nucleus. Proc Natl Acad Sci USA 2010 14 17421 17426 10.1073/pnas.1008491107 20847293 PMC2951462

[B23] Stam R Jupe J Howden AJM Morris JA Boevink PC Hedley PE Huitema E Identification and characterisation of CRN effectors in Phytophthora capsici shows modularity and functional diversity. PLOS ONE 2013 14 e59517 10.1371/journal.pone.0059517 23536880 PMC3607596

[B24] Tan MYA Hutten RB Visser RF Eck H The effect of pyramiding Phytophthora infestans resistance genes R Pi-mcd1 and R Pi-ber in potato. Theor Appl Genet 2010 14 117 125 10.1007/s00122-010-1295-8 20204320 PMC2871099

[B25] Zhu S Li Y Vossen J Visser RF Jacobsen E Functional stacking of three resistance genes against Phytophthora infestans in potato. Transgenic Res 2012 14 89 99 10.1007/s11248-011-9510-1 21479829 PMC3264857

[B26] Jupe F Pritchard L Etherington G MacKenzie K Cock P Wright F Sharma SK Bolser D Bryan G Jones J Hein I Identification and localisation of the NB-LRR gene family within the potato genome. BMC Genomics 2012 14 75 10.1186/1471-2164-13-75 22336098 PMC3297505

[B27] Fry WE Phytophthora infestans: the plant (and R gene) destroyer. Mol Plant Pathol 2008 14 385 402 10.1111/j.1364-3703.2007.00465.x 18705878 PMC6640234

[B28] Ballvora A Ercolano MR Weiß J Meksem K Bormann CA Oberhagemann P Salamini F Gebhardt C The R1 gene for potato resistance to late blight (Phytophthora infestans) belongs to the leucine zipper/NBS/LRR class of plant resistance genes. Plant J 2002 14 361 371 10.1046/j.1365-313X.2001.01292.x 12000683

[B29] Champouret N Bouwmeester K Rietman H van der Lee T Maliepaard C Heupink A van de Vondervoort PJI Jacobsen E Visser RGF van der Vossen EAG Govers F Vleeshouwers VGAA Phytophthora infestans isolates lacking class I ipiO Variants are virulent on Rpi-blb1 potato. Mol Plant Microbe Interact 2009 14 1535 1545 10.1094/MPMI-22-12-1535 19888819

[B30] Avrova AO Boevink PC Young V Grenville-Briggs LJ van West P Birch PR Whisson SC A novel Phytophthora infestans haustorium-specific membrane protein is required for infection of potato. Cell Microbiol 2008 14 2271 2284 10.1111/j.1462-5822.2008.01206.x 18637942

[B31] Kanneganti TD Huitema E Cakir C Kamoun S Synergistic interactions of the plant cell death pathways induced by *Phytophthora infestans *Nep1-like protein PiNPP1.1 and INF1 elicitin. Mol Plant Microbe Interact 2006 14 854 863 10.1094/MPMI-19-0854 16903351

[B32] Qutob D Kamoun S Gijzen M Expression of a *Phytophthora sojae *necrosis-inducing protein occurs during transition from biotrophy to necrotrophy. Plant J 2002 14 361 373 10.1046/j.1365-313X.2002.01439.x 12410814

[B33] Ah Fong AM Judelson HS Cell cycle regulator Cdc14 is expressed during sporulation but not hyphal growth in the fungus-like oomycete *Phytophthora infestans*. Mol Microbiol 2003 14 487 494 10.1046/j.1365-2958.2003.03735.x 14617173

[B34] Randall TA Dwyer RA Huitema E Beyer K Cvitanich C Kelkar H Ah Fong AMV Gates K Roberts S Yatzkan E Gaffney T Law M Testa A Torto T Zhang M Zheng L Mueller E Windass J Binder A Birch PRJ Gisi U Govers F Gow N Mauch F van West P Waugh M Yu J Boller T Kamoun S Lam ST Judelson HS Large-scale gene discovery in the oomycete *Phytophthora infestans *reveals likely components of phytopathogenicity shared with true fungi. Mol Plant Microbe Interact 2005 14 229 243 10.1094/MPMI-18-0229 15782637

[B35] consortium sg The tomato genome sequence provides insights into fleshy fruit evolution. Nature 2012 14 635 641 10.1038/nature11119 22660326 PMC3378239

[B36] Yamaguchi Y Pearce G Ryan CA The cell surface leucine-rich repeat receptor for AtPep1, an endogenous peptide elicitor in Arabidopsis, is functional in transgenic tobacco cells. Proc Natl Acad Sci USA 2006 14 10104 10109 10.1073/pnas.0603729103 16785433 PMC1502513

[B37] Moy P Qutob D Chapman BP Atkinson I Gijzen M Patterns of gene expression upon infection of soybean plants by *Phytophthora sojae*. Mol Plant Microbe Interact 2004 14 1051 1062 10.1094/MPMI.2004.17.10.1051 15497398

[B38] Qutob D Kemmerling B Brunner F Kufner I Engelhardt S Gust AA Luberacki B Seitz HU Stahl D Rauhut T Glawischnig E Schween G Lacombe B Watanabe N Lam E Schlichting R Scheel D Nau K Dodt G Hubert D Gijzen M Nurnberger T Phytotoxicity and innate immune responses induced by NEP1-like proteins. Plant Cell 2006 14 3721 3744 10.1105/tpc.106.044180 17194768 PMC1785393

[B39] Grenville-Briggs LJ Avrova AO Bruce CR Williams A Whisson SC Birch PR van West P Elevated amino acid biosynthesis in Phytophthora infestans during appressorium formation and potato infection. Fungal Genet Biol 2005 14 244 256 10.1016/j.fgb.2004.11.009 15707845

[B40] Schlink K Down-regulation of defense genes and resource allocation into infected roots as factors for compatibility between Fagus sylvatica and Phytophthora citricola. Funct Integr Genomics 2009 14 253 264 19813036 10.1007/s10142-009-0143-x

[B41] Lopez-Gomez M Sandal N Stougaard J Boller T Interplay of flg22-induced defence responses and nodulation in Lotus japonicus. J Exp Bot 2012 14 393 401 10.1093/jxb/err291 21934117 PMC3245478

[B42] Dyrløv Bendtsen J Nielsen H von Heijne G Brunak S Improved Prediction of Signal Peptides: SignalP 3.0. J Mol Biol 2004 14 783 795 10.1016/j.jmb.2004.05.028 15223320

[B43] Krogh A Larsson Br von Heijne G Sonnhammer ELL Predicting transmembrane protein topology with a hidden markov model: application to complete genomes. J Mol Biol 2001 14 567 580 10.1006/jmbi.2000.4315 11152613

[B44] Altschul SF Gish W W M EW M DJ L Basic local alignment search tool. J Mol Biol 1990 14 403 410 2231712 10.1016/S0022-2836(05)80360-2

[B45] Thompson JD Higgins DG Gibson TJ Improved sensitivity of profile searches through the use of sequence weights and gap excision. Comput appl biosci: CABIOS 1994 14 19 29 8193951 10.1093/bioinformatics/10.1.19

[B46] Win J Morgan W Bos J Krasileva KV Cano LM Chaparro-Garcia A Ammar R Staskawicz BJ Kamoun S Adaptive evolution has targeted the C-terminal domain of the RXLR effectors of plant pathogenic oomycetes. Plant Cell 2007 14 2349 2369 10.1105/tpc.107.051037 17675403 PMC2002621

[B47] Bhattacharjee S Hiller NL Liolios K Win J Kanneganti TD Young C Kamoun S Haldar K The malarial host-targeting signal is conserved in the Irish potato famine pathogen. PLoS Pathog 2006 14 e50 10.1371/journal.ppat.0020050 16733545 PMC1464399

[B48] Du Z Zhou X Ling Y Zhang Z Su Z agriGO: a GO analysis toolkit for the agricultural community. Nucleic Acids Res 2010 14 W64 W70 10.1093/nar/gkq310 20435677 PMC2896167

[B49] VENNY. An interactive tool for comparing lists with Venn Diagrams. http://bioinfogp.cnb.csic.es/tools/venny/index.html

