# Supplementary figures and images for: Phytophthora capsici-tomato interaction features dramatic shifts in gene expression associated with a hemi-biotrophic lifestyle
Source: Genome Biol. 2013 Jun 25;14(6):R63. doi: 10.1186/gb-2013-14-6-r63 (PMC4054836; doi:10.1186/gb-2013-14-6-r63)

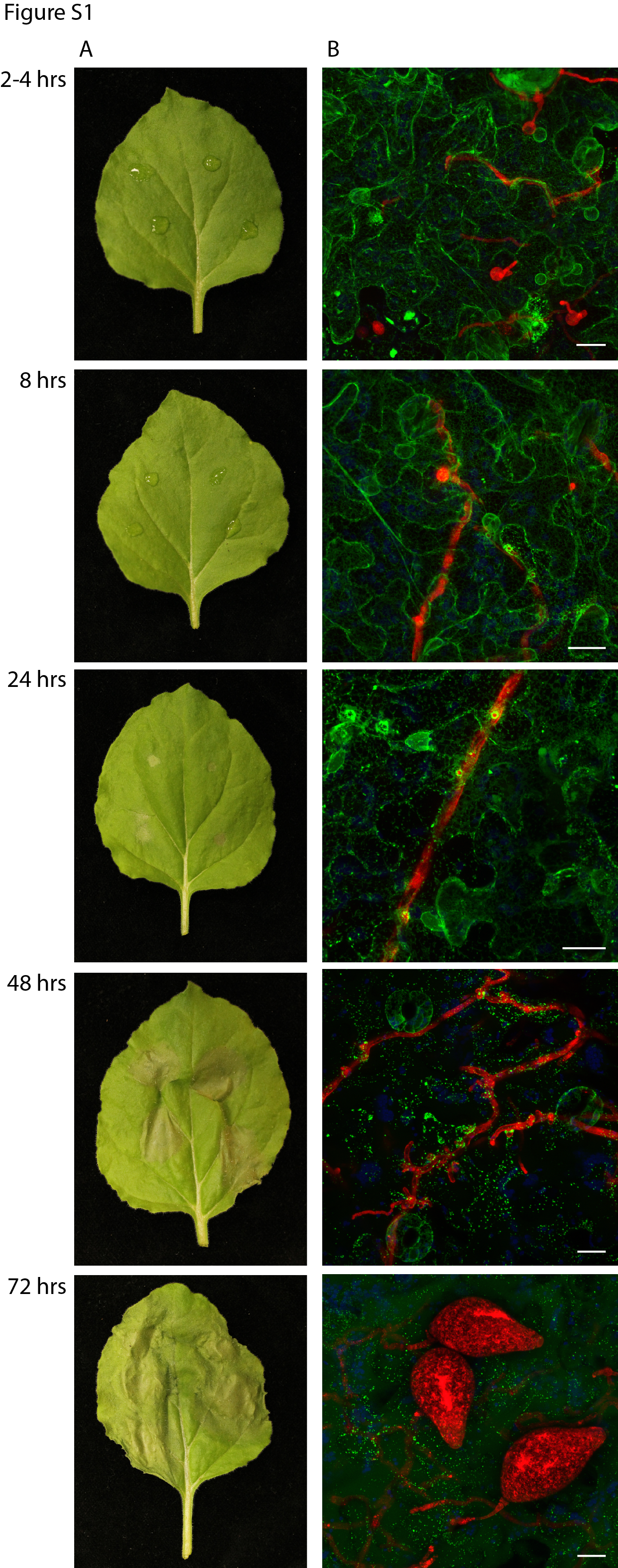

Supplement: Additional file 10 — Figure S1. Assessment of cell viability during Phytophthora capsici infection. Transgenic Nicotiana benthamiana plants constitutively expressing ER-eGFP, a green fluorescent protein (GFP) localized to the endoplasmic reticulum (ER) were used to assess whether host cells were alive during the course of infection. (A) Photographs of N. benthamiana leaves infected with zoospore suspensions of P. capsici at 0, 8, 24, 48, and 72 hpi. (B) Confocal microscopy images of N. benthamiana leaves infected with a transgenic P. capsici strain expressing the fluorescent protein TdTomato. Within the first 24 hours, the host ER was largely intact despite the presence of P. capsici, and haustoria were often seen to invaginate living cells. After 24 hours, the ER network was disrupted as shown by the unstructured distribution of GFP, suggesting dead or dying cells. Bar = 20 μm. [file gb-2013-14-6-r63-S10.PNG]

ni 0 8 16 24 48 72

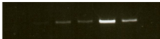

*PcHmp1*

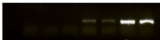

*PcNpp1*

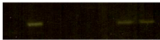

*PcCdc14*

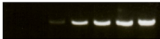

*PcTub*

Supplement: Additional file 11 — Figure S2. Reverse transcription PCR verification of marker-gene expression during infection. Expression of the marker genes PcHmp1, PcNpp1, PcCdc14, and PcTub (constitutive control) was tested by semi-quantitative PCR on cDNA derived from a time-course infection series used for the microarrays. Amplification of genes on cDNA derived from water-inoculated control (non-infected; ni) and tomato harvested 0, 8, 16, 24, 48, and 72 hpi with Phytophthora capsici. [file gb-2013-14-6-r63-S11.PDF]
